# The Effect of Flat Dual-Cure Stabilizer Occlusal Splint in Pain Relief of Individuals Suffering from Migraine Headaches

**DOI:** 10.2174/1874210601812010501

**Published:** 2018-07-31

**Authors:** Majid Sahebi, Somayeh Zeighami, Mohammadreza Hajimahmoudi

**Affiliations:** Dental Research Center, Dentistry Research Institute and Department of Prosthodontics, School of Dentistry, Tehran University of Medical Sciences, North Kargar Street, Enghelab Square, Tehran, Iran

**Keywords:** Occlusal splint, Migraine disorders, Headache, Visual analog scale, Flat Dual-Cure Stabilizer, International Classification of Headache Disorders (ICDH)

## Abstract

**Background:**

No study on the effect of dual-cure stabilizer splint without canine ramp in migraine patients is present.

**Objective:**

This study was conducted to determine the effects of maxillary flat dual-cure stabilizer occlusal splint on severity, frequency and episodes of headaches in individuals suffering from a migraine.

**Methods:**

In this interventional clinical trial, 30 eligible patients were divided into 2 groups (case and control); each group consisted of 8 men and 7 women. Dual-cure stabilizer splint was made for patients in the case group and they used the adjusted splint 20 hours a day for 6 weeks. The severity, frequency and episodes of migraine attacks before and after using the splint were determined. For grading pain, severity visual analogue scale was used. The data were analyzed using *SPSS* 20 and Kolmogorov-Smirnov test and paired t-test.

**Results:**

The severity, frequency, and episodes of migraine attacks before and after using the splint were reduced by 56%, 68%, and 72%, respectively. The reduction was statistically significant (*p* < 0.05).

**Discussion:**

Despite the effect of occlusal devices on the migraine disorder is controversial, the most researchers agree that using these appliances can be effective in reducing headache in migraine patients.

**Conclusion:**

Given the favorable effects of dual-cure stabilizer splint on reducing the severity, frequency and episodes of migraine headaches, the device can be used as an effective alternative therapy besides common pain-relieving methods.

## 1 INTRODUCTION

A migraine is a common progressive disorder. Migraineurs experience severe pain and more than 90% of them report substantial disability during their headaches. From a clinical aspect, the most common comorbidities of migraine are anxiety disorders and depression. The 3 best features that have predictive value for migraine are nausea, disability, and photophobia. Actually, if the patient reports 2 of these 3 symptoms, they have an 81% probability of having migraine. For patients with 3 symptoms, this probability according to the International Headache Society (IHS) diagnostic criteria for migraine is 93% [1]. According to the criteria of the International Classification of Headache Disorders (ICDH), migraine headaches are divided into two categories: episodic and chronic [2].

The correlation between migraine headaches and pericranial muscle spasm has been clearly established [3, 4]. The electromyographic studies also showed the increased electrical activity of pericranial muscles at the incidence of common migraine attacks in case groups [4, 5]. Moreover, jaw disorders have been observed mostly in patients with migraine headaches. These patients mostly experience muscular fatigue in masticatory muscles and sounds of temporomandibular joint [6, 7].

A migraine is treated using medical and non-medical methods. One of the non-medical methods is using occlusal splints. Using the stabilizer splint seems to have following changes: Situating the teeth in the centric relation, helping the facial muscles to be in the state of relaxation, equilibrium and stability in the centric relation and establishing a consistent contact between mandibular and maxillary teeth [8, 9].

Furthermore, other researchers showed a decrease in the number and severity of headache attacks following a functional treatment combined with exercise therapy, splint, and correction of occlusion [10, 11].

Moreover, the effects of the occlusal treatment in patients with various types of headache have been examined, and the patients showed a decrease in the number and severity of headache attacks [12, 13]. The use of splint therapy alone has also been recommended as an appropriate treatment for pain relief by researchers [4, 14, 15].

Shankland compared the effect of using the pericranial suppression mechanism with that of occlusal splints on reducing the frequency and severity of migraine headaches [16]. The study measured the effect of Nociceptive Trigeminal Inhibition Suppression System (NTI) *versus* the effects of occlusal splints. This appliance is made in the centric occlusion, and no other determinant such as the anterior guide is added to the occlusion. After 8 weeks of using NTI system, 16% of patients showed a decrease in the incidence of migraine headaches by 85-100%, and only 7% of patients using splints showed 85-100% decrease in the incidence of migraine headaches.

Mitrirattanakul and Merrill examined the effects of headaches on patients with oral and facial pain. They found that the concurrent primary headaches and musculoskeletal disorders might increase the headache disability in patients, and clinicians should treat both diseases simultaneously in order to achieve favorable results [17].

Wright *et al*. studied the relief of headache symptoms after using the stabilization appliance and self-medication methods in patients going to a clinic for chronic headaches. They concluded that the use of the appliance with self-medication without any association with the type of headache (tension, migraine with aura, and migraine without aura) was useful in many patients suffering headaches [18].

Juehring *et al*. analyzed the results of treatment using Vojta/Dynamic Neuromuscular Stabilization device for 12 weeks in a 49-year-old woman with chronic migraine headaches. The study showed a decrease in frequency, duration, and severity of the headaches after using the stabilization device [19]. Numerous studies tried to treat or significantly reduce migraine headaches through correction of occlusion using a hard acrylic splint [3, 4]. According to our knowledge, there is no study about the effect of dual-cure stabilizer splint without canine ramp in migraine patients. The present study was conducted to determine the effect of the dual-cure stabilizer splint in reducing the pain of migraine patients.

## 2 MATERIALS AND METHODS

This randomized non blinded clinical trial was designed on patients, from March 2015 to October 2016, going to the neurology ward of teaching hospitals and then referred to the Department of Tempro Mandibular Joint (TMJ), School of Dentistry, Tehran University of Medical Sciences, Tehran, Iran. This study has been registered as thesis no 4945 in dental school and an ethical board approved the design of the study.

### 2.1 Sample Size Calculation

The sample size was calculated by the following formula and information from similar paper [20].

(1) $$n=\frac{(\partial_{1}^{2}+\partial_{2}^{2})(z_{\alpha }+z_{\beta }{)}^{2}}{(\mu_{1}-\mu_{2}{)}^{2}}$$

∂_1_: Standard deviation of pain severity in control group= 0.5

∂_2_: Standard deviation of pain severity in case group= 0.22

µ_1_: Mean of pain severity in control group= 2.02

µ_2_: Mean of pain severity in case group= 1.28

α<0.1

β < 0.05

The sample size was 15 in each group (case and control). Patients were selected by purposive sampling and divided into two groups by method of random blocks.

### 2.2 Patient Selection Criteria

The inclusion and exclusion criteria are shown in Table **1**. The inclusion criteria were: patient’s age between 20-55 years, good oral hygiene without periodontal problem, headache confirmed as migraine disorder by a neurologist, no history of treatments associated to the occlusion (including the splint therapy), having all the teeth or maximum one extracted tooth (except wisdom teeth), having maximum one bridge or three laminated teeth, having received at least two standard treatment for episodic migraine HA (acetaminophen (325 mg), diazepam (2 mg), and tripheloprazine (1 mg), sublingual ergotamine, dihydroergotamine injection, or ergotamine-C tablets).

The patients were excluded if they had any history of using opiate, medication or drug that could jeopardize the treatment outcome, systemic diseases increasing or decreasing the pain threshold (*e.g.,* fibromyalgia), a source of pain other than migraine in their head and neck (including abscess, tooth decay, and impacted teeth), a history of diseases or conditions overlapping with migraine headache (*e.g*., head and neck trauma or stroke), signs of untreated periodontal disease or other mucosal and bone lesions, gingival recession or periodontal pockets (with an endodontic or periodontal origin).

Thirty patients (16 males and 14 females) were included in this study and signed an informed consent form after being informed about the treatment protocol. The treatment period for patients was determined 6 weeks.

### 2.3 Before Treatment

**Fig. (1):**
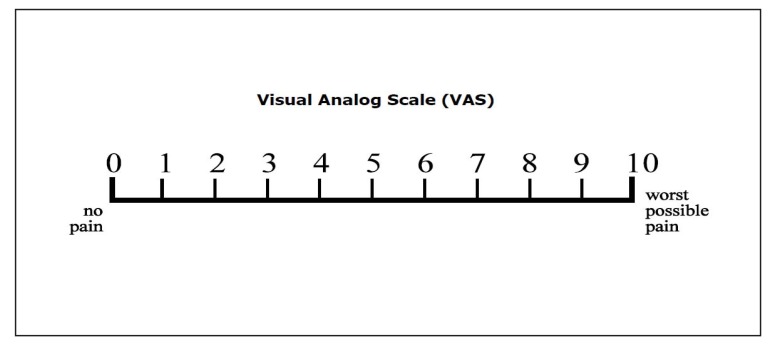
VAS diagram.

Each patient filled a questionnaire on the history of headache attacks. The main questions included the following: how many years do you have migraine disorder? how much is the headache severity according to VAS? how many headaches do you have in one month? how long does each headache last?. The pain severity was scored according to the Visual Analogue Scale (VAS) from 0 (no pain) to 10 (worst possible pain) [21] (Fig. **1**). Each patient received daily checklists for 6 weeks. The patients were asked to record the starting and ending time of the pain and severity of the pain.

### 2.4 Treatment Procedure

The patients in the control group were left on their same baseline medications and the patients in the case group received the dual-cure stabilizer occlusal splint without canine ramp on the maxilla. For making a splint for each patient, impressions were made using irreversible hydrocolloid (Alginoplast, Heraeus Kulzer GmbH & Co., Wehrheim, Germany) and patients’ bite in centric relation (using Dawson’s bimanual technique) was recorded with base plate wax (Dentsply, Weybridge, United Kingdom).

In the delivery session, appliances were adjusted to have equal contact on the anterior and posterior teeth in centric relation. Also, patients were educated to use the appliance all the day except for eating and cleaning.

### 2.5 Patient Recall

The patients in the case group were recalled two weeks later (first call). They were re-evaluated in terms of retention and stability, the shape of the splint, and occlusal contacts because the presence of these items led to patients’ comfort and treatment continuity and stability.

The next meeting was held at the end of the sixth week (second call) to make final adjustments, submit checklists, and complete the questionnaire again. The All patients (the control and the case group) were asked to bring their daily checklist and complete the questionnaire again. Moreover, the case group was instructed how to reduce the time of using splint, as the time of using splint was reduced by 8 hours at the beginning of the seventh week and since then reduced daily by one hour, as the splint should be used only at nights in the eighth week [22]. All splints made by an expert technician and the study was down by a blinded prosthodontist. The data were analyzed using *SPSS* 20 (*SPSS* Inc., IL, USA) and Kolmogorov-Smirnov test and paired t-test. Level of significance was 0.05.

## 3 RESULTS

The severity, frequency and episodes of migraine attacks before and 6 weeks after using the splint were determined.

Most patients were 20-30 years old. Given the confidence interval of 95%, mean pain severity in patients using the splint was lower (3.5-5) than that in the control group [4-6]. Mean frequency of pain in patients using the splint was lower and shorter (1-2) than that in the control group (2.5-4). Mean duration of pain episode, in patients using the splint was shorter (2.5-5) than that in the control group (7.5-13).

Based on the results of Kolmogorov-Smirnov test, all the studied variables followed a normal distribution in the statistical population (*P*-value ≥ 0.05) (Tables **2**, **3**). The measurements in the control group before and after the treatment were similar. There was a significant difference between results of case group before and after the treatment in terms of all the variables (*p* < 0.05). Patients using the splint (as an alternative treatment) showed a tangible reduction in all the variables after the treatment. The results of study have been summarized in Tables **2**, **3** and **4**. Demographic information of case group has summarized in Table **5**.

## 4 DISCUSSION

In the present study, the effect of dual-cure stabilizer splint without canine ramp on reducing the pain of migraine headaches was studied.

According to the results of this study, the dual-cure stabilizer splint could considerably reduce pain severity, frequency, and duration of pain episode in patients with migraine headaches. The severity, frequency, and duration of pain episode of migraine attacks before and after using the splint were reduced by 55.8%, 67.7%, and 72.1%, respectively.

Despite the different ideas of researchers and dentists about the use of splints for treating migraine headaches, the dominant opinion is that the use of different types of the splint can be effective in reduction of migraine headaches [23, 24].

The previous studies showed that despite the diversity and differences in the designs of different splints, these appliances enjoyed special medical efficiency [25]. However, the therapeutic mechanism of occlusal splints has not been accurately determined [26]. Many diversities have been reported in relation to the details of flat-plane occlusal splints with [27] and without canine ramp [28].

After adjusting the splint with the mouth, the associated muscles would rest, teeth would be protected against adverse effects of parafunction, and periodontal ligament movements in teeth would become normal. Moreover, these splints can conduct condyles and jaws to the centric relation [29].

It seems that the presence of a foreign body in the mouth may reduce jaws’ nocturnal activity due to the changes made in oral tactile stimuli, size of mouth, and the space needed for the tongue [30]. Using the oral splint, patients may be aware of the situation and probable adverse effects of jaws’ movements, which is known as cognitive awareness [31]. One may argue that the mechanism is efficient only in the daily use of splints, while, regarding the claims for positive placebo effects of splints [23], the argument is not true any longer, and the positive effects of splints have been also proved for nights. In this respect, the use of splints with various designs does not lead to significantly different therapeutic results, and all the designs have been successful in achieving the desired results.

Carraro and Caffesse studied TMJ patients’ responses to the stabilization splint and showed that 82% of them responded to the treatment with splints favorably and their symptoms of TMJ and muscular pain and headaches were treated [32]. Results of the above study agreed with those of the present study in this regard.

Dao *et al*. studied the therapeutic effects of oral splints using a randomized controlled blind parallel design and showed that patients’ scores for pain were significantly reduced following the use of splints [33]. Shankland also compared the effect of using a device with pericranial suppression mechanism with that of occlusal splints in terms of reduction of the frequency and severity of migraine headaches. Shankland found that the incidence of migraine headaches reduced by 85-100% in 16% of patients using the device and 7% of patients using splints [16]. Jokstad studied the clinical effects of two different designs of splints on the treatment of temporomandibular disorders and showed that mean VAS scores of headache and pain caused by temporomandibular disorders reduced within the 3 months [34].

Furthermore, Lamey and Barclay treated patients with a classic migraine using acrylic splints and reported a significant reduction in number and severity of migraine attacks [35]. Another study treated patients with disc displacement using a forwarding splint and reported an obvious reduction in temporal pain, pain in the ear and in front of the ear, the TMJ’s sensitivity and pain, and mouth locking [36]. Quayle *et al*. treated patients with a migraine, tension, and tension-vascular headache using splints and showed that a considerable number of patients were satisfied with the treatment after 3 months although a tension headache in most of the patients did not decrease significantly [37]. Lamey studied the effects of using acrylic splints on migraine headaches and showed that the number of headache attacks in patients decreased by 40% after using the splint [38]. In the present study, the frequency of headaches (monthly) was reduced by 68.5% after a 6-week use of dual-cure splints, and the reduction was higher than that reported in the above study.

Although a different design of splints was used in the present study to reduce patients’ migraine headaches, the obtained results on the reduced severity, frequency, and duration of pain episode conformed to and was even more favorable than, those of the previous studies.

The limitation of this study was that there was no group with a standard occlusal splint to compare with dual-cure stabilizer splint without canine ramp group. Also to achieve more conclusive and definitive results, it is better to design a study with more patient and more follow up time.

It is recommended that future clinical studies be conducted to compare occlusal splints with different designs.

## CONCLUSION

Under limitation of this study, the following conclusions were obtained:

1. Dual-cure stabilizer splint without canine ramp reduced the severity, frequency, and episodes of migraine attacks before and after using the splint.
2. The severity of headaches decreased by more than fifty percent.
3. The frequency of headache was more than 4 times a month and now after treatment is less than twice a month.
4. The duration of each headache decreased by more than 10 hours.

## Figures and Tables

**Table 1:** Inclusion and exclusion criteria.

Inclusion Criteria
1. Age between 20-55 years 2. Good oral hygiene without periodontal problem 3. Headache confirmed as migraine disorder by a neurologist 4. No history of treatments associated to the occlusion (including the splint therapy) 5. Having all the teeth or maximum one extracted tooth (except wisdom teeth) 6. Having no more than one bridge or three laminated teeth 7. Having received at least two standard treatment for episodic migraine headache (acetaminophen (325 mg), diazepam (2 mg), and tripheloprazine (1 mg), sublingual ergotamine, dihydroergotamine injection, or ergotamine-C tablets)
**Exclusion Criteria**
1. History of using opiate, medication or drug that could jeopardize the treatment outcome 2. Systemic diseases increasing or decreasing the pain threshold (*e.g*., fibromyalgia) 3. A source of pain other than migraine in their head and neck (including abscess, tooth decay, and impacted teeth) 4. History of diseases or conditions overlapping with migraine headache (*e.g*., head and neck trauma or stroke) 5. Signs of untreated periodontal disease or other mucosal and bone lesions 7. Gingival recession or periodontal pockets (with an endodontic or periodontal origin)

**Table 2:** Results obtained from Kolmogorov-Smirnov test about the studied variables.

Group	Statistical Specifications	Pain Severity (VAS)		Frequency of Pain (per month)		Pain Episodes (hour)	
		Before Treatment	After Treatment	Before Treatment	After Treatment	Before Treatment	After Treatment
Case	Mean ± SD	8.9±1.1	4±1.3	4.7±0.1	1.4±0.5	14.6±5.6	3.9±1.8
	Z-score	1	0.9	0.9	1.4	0.6	0.7
	*P*-value	0.3	0.5	0.4	0.05	0.8	0.7
Control	Mean ± SD	5±1.3	5±1.3	3.2±1.0	3.2±1.0	10.7±4.4	10.7±4.4
	Z-score	1.2	1.2	0.9	0.9	0.5	0.5
	*P*-value	0.1	0.1	0.3	0.3	0.9	0.9

**Table 3:** Paired t-test results for the studied variables in case group.

Statistical Specifications	The Difference between Pain Severity before and after Treatment (VAS)	The Difference between Frequency of Pain before and after Treatment (per month)	The Difference between Pain Episodes before and after Treatment (hour)
Mean ± SD	-10.7 ± 4.4	-3.2 ± 1.0	-5 ± 1.3
Z-score	-8.3	-12.2	-14.8
P-value	0.0001	0.0001	0.0001

**Table 4:** Reduction percent in studied variables in case group.

Variable	Mean ± SD(%)	Confidence Interval of 95%
Pain severity	55.8±11.5	49.4-62.2
Frequency of pain	67.7±12.16	60.9-74.4
Pain episodes	72.1 ± 10.8	65.3-78.9

**Table 5:** Demographic information of case group before and after treatment.

Gender	Mean age of Patients (year)	Duration of Headache History (year)	Mean Headache Severity (VAS)		Mean Frequency of Headache (per month)		Mean Duration of Each Headache (hour)	
			Before Treatment	After Treatment	Before Treatment	After Treatment	Before Treatment	After Treatment
Male (n=8)	37.9	10.3	8.6	4.2	4.6	1.5	14.7	4
Female (n=7)	31.4	6.1	9.4	3.6	4.7	1.4	14.4	3.8

## References

[1] Lipton R.B., Bigal M.E. (2007). Ten lessons on the epidemiology of migraine.. Headache.

[2] Lipton R.B., Fanning K.M., Serrano D., Reed M.L., Cady R., Buse D.C. (2015). Ineffective acute treatment of episodic migraine is associated with new-onset chronic migraine.. Neurology.

[3] van Boxtel A., Goudswaard P. (1984). Absolute and proportional resting EMG levels in chronic headache patients in relation to the state of headache.. Headache.

[4] Tfelt-Hansen P., Lous I., Olesen J. (1981). Prevalence and significance of muscle tenderness during common migraine attacks.. Headache.

[5] Bakal D.A., Kaganov J.A. (1977). Muscle contraction and migraine headache: Psychophysiologic comparison.. Headache.

[6] Forssell H., Kangasniemi P. (1984). Mandibular dysfunction in patients with migraine.. Proc. Finn. Dent. Soc..

[7] Lous I., Olesen J. (1982). Evaluation of pericranial tenderness and oral function in patients with common migraine, muscle contraction headache and ‘combination headache’.. Pain.

[8] Barnes M.F., Geary J.L., Clifford T.J., Lamey P.J. (2006). Fitting acrylic occlusal splints and an experimental laminated appliance used in migraine prevention therapy.. Br. Dent. J..

[9] Lapeer G.L. (1980). Reduction of the painful sequelae of migraine headache by use of the occlusal diagnostic splint: An hypothesis.. Cranio.

[10] Magnusson T., Carlsson G.E. (1980). Changes in recurrent headaches and mandibular dysfunction after various types of dental treatment.. Acta Odontol. Scand..

[11] Magnusson T., Carlsson G.E.A. (1983). A 21/2-year follow-up of changes in headache and mandibular dysfunction after stomatognathic treatment.. J. Prosthet. Dent..

[12] Forssell H., Kirveskari P., Kangasniemi P. (1987). Response to occlusal treatment in headache patients previously treated by mock occlusal adjustment.. Acta Odontol. Scand..

[13] Forssell H., Kirveskari P., Kangasniemi P. (1985). Changes in headache after treatment of mandibular dysfunction.. Cephalalgia.

[14] Kemper J.T., Okeson J.P. (1983). Craniomandibular disorders and headaches.. J. Prosthet. Dent..

[15] Ahlin J.H., Atkins G. (1984). A screening procedure for differentiating temporomandibular joint related headache.. Headache.

[16] Shankland W.E. (2001). Migraine and tension-type headache reduction through pericranial muscular suppression: A preliminary report.. Cranio.

[17] Mitrirattanakul S., Merrill R.L. (2006). Headache impact in patients with orofacial pain.. J. Am. Dent. Assoc..

[18] Wright E., Anderson G., Schulte J. (1995). A randomized clinical trial of intraoral soft splints and palliative treatment for masticatory muscle pain.. J. Orofac. Pain.

[19] Juehring D.D., Barber M.R. (2011). A case study utilizing Vojta/Dynamic Neuromuscular stabilization therapy to control symptoms of a chronic migraine sufferer.. J. Bodyw. Mov. Ther..

[20] Glaros A.G., Owais Z., Lausten L. (2007). Reduction in parafunctional activity: A potential mechanism for the effectiveness of splint therapy.. J. Oral Rehabil..

[21] Warden V., Hurley A.C., Volicer L. (2003). Development and psychometric evaluation of the Pain Assessment in Advanced Dementia (PAINAD) scale.. J. Am. Med. Dir. Assoc..

[22] Dawson P. (2007). Functional occlusion from TMJ to smile design..

[23] Ekberg E., Vallon D., Nilner M. (2003). The efficacy of appliance therapy in patients with temporomandibular disorders of mainly myogenous origin. A randomized, controlled, short-term trial.. J. Orofac. Pain.

[24] Ekberg E., Nilner M. (2002). A 6- and 12-month follow-up of appliance therapy in TMD patients: A follow-up of a controlled trial.. Int. J. Prosthodont..

[25] Türp J.C., Komine F., Hugger A. (2004). Efficacy of stabilization splints for the management of patients with masticatory muscle pain: A qualitative systematic review.. Clin. Oral Investig..

[26] Kreiner M., Betancor E., Clark G.T. (2001). Occlusal stabilization appliances. Evidence of their efficacy.. J. Am. Dent. Assoc..

[27] Linde C., Isacsson G., Jonsson B.G. (1995). Outcome of 6-week treatment with transcutaneous electric nerve stimulation compared with splint on symptomatic temporomandibular joint disk displacement without reduction.. Acta Odontol. Scand..

[28] Lundh H., Westesson P.L., Eriksson L., Brooks S.L. (1992). Temporomandibular joint disk displacement without reduction. Treatment with flat occlusal splint *versus* no treatment.. Oral Surg. Oral Med. Oral Pathol..

[29] Dylina T.J. (2001). A common-sense approach to splint therapy.. J. Prosthet. Dent..

[30] Dubé C., Rompré P.H., Manzini C., Guitard F., de Grandmont P., Lavigne G.J. (2004). Quantitative polygraphic controlled study on efficacy and safety of oral splint devices in tooth-grinding subjects.. J. Dent. Res..

[31] Clark G.T., Beemsterboer P.L., Solberg W.K., Rugh J.D. (1979). Nocturnal electromyographic evaluation of myofascial pain dysfunction in patients undergoing occlusal splint therapy.. J. Am. Dent. Assoc..

[32] Carraro J.J., Caffesse R.G. (1978). Effect of occlusal splints on TMJ symptomatology.. J. Prosthet. Dent..

[33] Dao T.T., Lavigne G.J., Charbonneau A., Feine J.S., Lund J.P. (1994). The efficacy of oral splints in the treatment of myofascial pain of the jaw muscles: A controlled clinical trial.. Pain.

[34] Jokstad A., Mo A., Krogstad B.S. (2005). Clinical comparison between two different splint designs for temporomandibular disorder therapy.. Acta Odontol. Scand..

[35] Lamey P.J., Barclay S.C. (1987). Clinical effectiveness of occlusal splint therapy in patients with classical migraine.. Scott. Med. J..

[36] Tallents R.H., Katzberg R.W., Macher D.J., Roberts C.A. (1990). Use of protrusive splint therapy in anterior disk displacement of the temporomandibular joint: A 1- to 3-year follow-up.. J. Prosthet. Dent..

[37] Quayle A.A., Gray R.J., Metcalfe R.J., Guthrie E., Wastell D. (1990). Soft occlusal splint therapy in the treatment of migraine and other headaches.. J. Dent..

[38] Lamey P.J., Steele J.G., Aitchison T. (1996). Migraine: the effect of acrylic appliance design on clinical response.. Br. Dent. J..

